# Antimicrobial Peptides in Human Sepsis

**DOI:** 10.3389/fimmu.2015.00404

**Published:** 2015-08-20

**Authors:** Lukas Martin, Anne van Meegern, Sabine Doemming, Tobias Schuerholz

**Affiliations:** ^1^Department of Intensive Care and Intermediate Care, University Hospital RWTH Aachen, Aachen, Germany

**Keywords:** sepsis, antimicrobial peptides, synthetic, naturally occurring peptides, therapy

## Abstract

Nearly 100 years ago, antimicrobial peptides (AMPs) were identified as an important part of innate immunity. They exist in species from bacteria to mammals and can be isolated in body fluids and on surfaces constitutively or induced by inflammation. Defensins have anti-bacterial effects against Gram-positive and Gram-negative bacteria as well as anti-viral and anti-yeast effects. Human neutrophil peptides (HNP) 1–3 and human beta-defensins (HBDs) 1–3 are some of the most important defensins in humans. Recent studies have demonstrated higher levels of HNP 1–3 and HBD-2 in sepsis. The bactericidal/permeability-increasing protein (BPI) attenuates local inflammatory response and decreases systemic toxicity of endotoxins. Moreover, BPI might reflect the severity of organ dysfunction in sepsis. Elevated plasma lactoferrin is detected in patients with organ failure. HNP 1–3, lactoferrin, BPI, and heparin-binding protein are increased in sepsis. Human lactoferrin peptide 1–11 (hLF 1–11) possesses antimicrobial activity and modulates inflammation. The recombinant form of lactoferrin [talactoferrin alpha (TLF)] has been shown to decrease mortality in critically ill patients. A phase II/III study with TLF in sepsis did not confirm this result. The growing number of multiresistant bacteria is an ongoing problem in sepsis therapy. Furthermore, antibiotics are known to promote the liberation of pro-inflammatory cell components and thus augment the severity of sepsis. Compared to antibiotics, AMPs kill bacteria but also neutralize pathogenic factors such as lipopolysaccharide. The obstacle to applying naturally occurring AMPs is their high nephro- and neurotoxicity. Therefore, the challenge is to develop peptides to treat septic patients effectively without causing harm. This overview focuses on natural and synthetic AMPs in human and experimental sepsis and their potential to provide significant improvements in the treatment of critically ill with severe infections.

## Natural Occurrence in Humans – Change of AMPs in Inflammatory Disease

Antimicrobial peptides (AMPs) are an important component of multicellular organisms’ innate immune systems, targeting invading pathogens, including bacteria, viruses, fungi, and parasites (1). The growing relevance of AMPs in recent years is owed to their capability to overcome increasing antibiotic resistance due to their unique combination of anti-inflammatory, antimicrobial, and immunostimulatory qualities (2–4). Generally, AMPs differ greatly in sequence and structure. These peptides are predominantly short (10–50 amino acids) amphipathic molecules. Based on amino acid composition and secondary structures, they can be divided into four groups: (i) α-helical peptides, (ii) β-sheet peptides stabilized by two to four disulfide bonds, (iii) extended structures, and (iv) loop peptides with one disulfide bond (3, 5, 6).

The most extensively investigated peptides of the mammalian gene family are the defensins and cathelicidins. The defensins, consisting of the alpha and beta subgroups, represent more than 5% of the total protein of human neutrophils (7) and are derived from intestinal Paneth cells, neutrophils, macrophages (1), epithelial cells, mucosal epithelial cells, and keratinocytes (8). Through the stimulation of toll-like receptors (TLRs), including TLR-2, TLR-3, and TLR-5, α-defensins [human neutrophil peptides (HNP) 1–4] are released by their producing cells (4, 8–17). Altogether, six human α-defensins from the granules of neutrophils (HNPs1–4) and Paneth cells (human defensins including HD5 and HD6) as well as four human β-defensins derived from epithelial cells were studied in detail. It has been shown that alterations in expression may influence inflammatory disorders, which emphasizes the importance of these peptides in controlling and preventing microbial infections (18, 19). Some AMPs are constitutively expressed, whereas others can be induced [e.g., HNP 1–3, human beta-defensin (HBD)-2] in response to inflammation (8).

The mechanisms in neutrophil trafficking and function in sepsis have been reviewed previously in Ref. (20). A weakened response to chemotaxis and alterations in neutrophils may result after dysregulation of TLR expression. TLR activation itself results in a downstream liberation of AMP as well as cytokine and chemokine release. This, in turn, activates NF-kB and mitogen-activated protein kinase (MAPK) pathways. A constantly activated TLR may lead to a strongly increased expression of cytokines, thus aggravating sepsis in critically ill patients (20–22).

Cathelicidins are produced by proteolysis of the C-terminus of protein precursors. In humans, only one precursor, hCAP18, is produced mainly in leukocytes and epithelial cells and forms the LL-37 peptide, among others. The application of LL-37 in infection therapy has been hampered by its toxicity. The incubation of smooth muscle cells with 20 μM LL-37 resulted in 20-fold higher DNA fragmentation compared to the control (23). Moreover, human serum inhibited the antimicrobial effects. Another problem is that some multiresistant strains (e.g., USA600-MRSA) showed increased resistance against LL-37, which has been suggested to be responsible for higher mortality rates (24). When the *N*-terminal hydrophobic amino acids of LL-37 were removed, a decrease in cytotoxicity was detected. Furthermore, inhibition of the antimicrobial and lipopolysaccharide (LPS)-neutralizing effects of LL-37 by human serum was reduced. Thus, LL-37-derived peptides may provide a benefit when treating sepsis patients (23). Innate immunity, especially in the case of sepsis, may be influenced by vitamin D status. In turn, vitamin D status regulates the LL-37 levels in sepsis (25). The possible underlying mechanism has been described as a TLR activation of macrophages, which results in increased expression of both the receptor and the hydroxylase of vitamin D, thus inducing AMPs (26). Interestingly, a deficiency in vitamin D is a predictor of sepsis in critically ill patients and results in higher mortality in the intensive care unit (ICU) (27).

Bactericidal/permeability-increasing protein (BPI) is an AMP stored in leukocytes that has a high affinity for LPSs of Gram-negative bacteria. The anti-infective properties of BPI include the permeabilization of bacterial membranes, in addition to the neutralization of LPS (28).

Lactoferrin is a glycoprotein located in the majority of exocrine secretions (e.g., milk, tears, nasal secretions) (29) and in neutrophils (30). Its wide antimicrobial spectrum supports the body’s immune response to bacterial, viral, and fungal pathogens (31–34). Anti-bacterial peptides, which are part of the polypeptide chain of lactoferrin, are released after proteolysis and may be developed for new agents in antimicrobial therapy (35, 36). Nevertheless, the exact mechanism of the antimicrobial activity of lactoferrin peptides has not been described to date.

**Figure 1:**
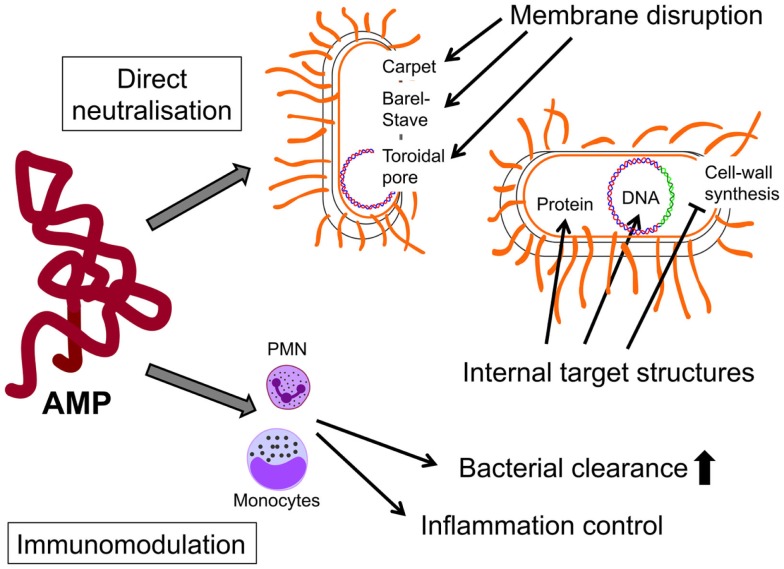
**Different modes of action of antimicrobial peptides**. AMPs may have direct neutralizing effects on bacteria e.g., by membrane disruption through pore forming or by targeting internal structures of bacteria. In addition to direct effects, AMPs may modulate cells of the adaptive immunity (neutrophils, t-cells, macrophages) to control inflammation and/or to increase bacterial clearance. Modified from Ref. (3). AMPs, antimicrobial peptides.

**Figure 2:**
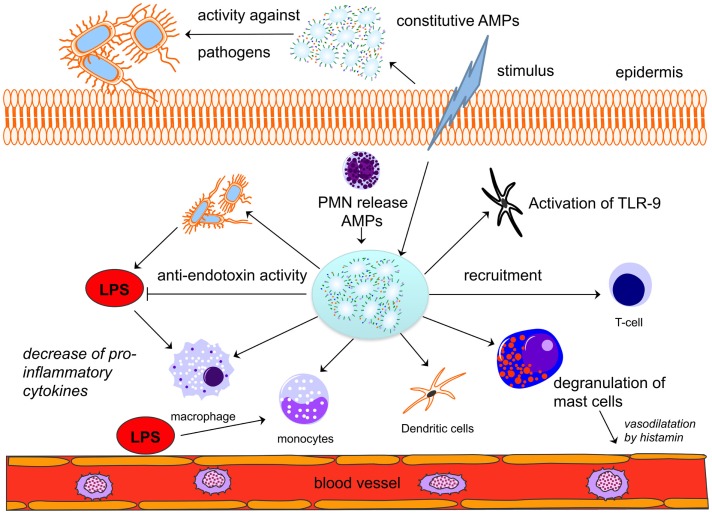
**Antimicrobial peptides play a central role in innate and adaptive immunity**. A given stimulus by bacteria leads to the release of constitutively expressed AMPs in different cells (here: epidermis). AMPs are released by neutrophils and will activate and recruit macrophages, monocytes, dendritic cells, and T-cells. A direct anti-endotoxin effect of AMPs may decrease the activation of immune cells and thus lead to a decrease in pro-inflammatory cytokine release. Modified from Ref. (26). AMPs, antimicrobial peptides; LPS, lipopolysaccharide; PMNs, neutrophils.

Antimicrobial peptides prevent and control microbial infections by both direct antimicrobial killing and innate immune modulation (4, 9, 37, 38). Direct antimicrobial killing is achieved by the disruption of bacterial cell membranes or translocation into bacteria to affect internal targets (3) (Figure 1). The cationic amphipathic AMPs bind to the negatively charged phospholipids of the bacterial cell membranes (3). It is assumed that pore formation and non-specific membrane permeabilization lead to a disruption of the membrane (Figure 1). Recently, there has been increasing evidence that molecules on the cell surface act as targets for the AMPs and induce direct killing (10, 39). However, the immunostimulatory properties have been more appreciated for their diversity, including cell migration, survival and proliferation, induction of antimicrobial and immune mediators such as cytokines/chemokines, wound healing, and angiogenesis (Figure 2) (4, 40). The results show that HBD-2 seems to be chemotactic for cells that express the human chemokine receptor CCR6 (41). CCR6 is preferentially expressed by immature monocytic dendritic cells (DCs) and CD8 + T-cells that have the memory phenotype (42). Additionally, HNP 1–3 are chemotactic for monocytes, immature DCs and CD4+ and CD8+ T-cells (43, 44). Furthermore, they induce the release of pro-inflammatory cytokines such as IFN-λ, IL-6, and IL-10 from T-cells as well as TNF-α and IL-1β from monocytes (45, 46). These processes contribute to the maturation of DCs, which link the innate and adaptive immune systems. Stimulation with TNFα contributes to this maturation. DCs activate CD4+ T-cells (and their subsets) and CD8+ T-cells as well as B-cells. In turn, monocytes may be induced by a peptide to differentiate into DCs (47).

Moreover, AMPs protect the organism against harmful pro-inflammatory immune responses, especially against TLR-induced cytokine release. For instance, the above described LL-37 allows free DNA fragments to enter DCs. Consequently, IFN-α is released in reasonable amounts by TLR-9 interaction (Figure 2) (48). The free DNA fragments are able to neutralize extracellular LPS and/or stimulate the expression of anti-inflammatory mediators by affecting different signaling pathways associated with, for example, MyD88 and TRIF (49–52).

## Role of AMPs in Sepsis

Antimicrobial peptides were identified nearly 100 years ago in body fluids and on body surfaces after inflammatory stimulation (53). However, studies of AMPs in patients with severe sepsis or septic shock are limited. AMPs have been investigated in patients with abscesses, peritonitis, or uninfected body fluid levels of the LPS-binding protein (LBP) and BPI, which prevents endotoxin binding to CD14. The BPI/LBP ratio was significantly elevated in abscesses compared to peritoneal and non-infected fluids. Moreover, the BPI concentration was higher in abscesses with Gram-positive compared to those with Gram-negative organisms. The authors concluded that BPI might attenuate the local inflammatory response and the systemic toxicity of endotoxins released during Gram-negative infections (54). A further study in the same year investigated the levels of polymorphonuclear leukocyte surface BPI, plasma BPI, and plasma LBP in normal human volunteers who were administered *Escherichia coli* LPS and in patients with sepsis and Gram-negative infections. Compared with controls, LPS-challenged volunteers and patients with sepsis both exhibited increased concentrations of polymorphonuclear leukocyte surface BPI and plasma LBP (55). Rintala et al. investigated BPI levels and BPI/neutrophil ratios in 42 healthy controls and 34 patients with severe sepsis. Because of an association between decreased arterial blood pressure and levels of BPI, the authors concluded that BPI might indicate the severity of organ dysfunction in sepsis (56).

As endogenous ligands of TLR-4, HBD 1–3 interact with TLR-4 on immune cells and regulate the expression of inflammatory mediators via the NF-κB pathway (57).

A study determined concentrations of HBD-1, HBD-2, and cathelicidin LL-37/hCAP-18 in tracheal aspirates of mechanically ventilated newborn infants. Concentrations of AMPs correlated with each other and with levels of interleukin-8 and tumor necrosis factor-α in the bronchoalveolar lavage fluid. Pulmonary or systemic infections were associated with significantly increased concentrations of HBD-1, HBD-2, and LL-37 (58). A further study investigated the effect of overexpression of BD-2 on lung injury to evaluate whether the function of BD-2 in the lung could be attributed to both antimicrobial action and modulation of the immune response. Therefore, recombinant adenoviruses carrying an expression cassette of rat BD-2 or control adenovirus carrying an empty vector were administered intratracheally to Sprague-Dawley rats. After 48 h, acute lung injury was induced by either *Pseudomonas aeruginosa* infection or cecal ligation and puncture (CLP). The amounts of the *P. aeruginosa* in the lung with BD-2 overexpression were significantly lower compared to those of the controls. Furthermore, the overexpression of BD-2 reduced alveolar damage and interstitial edema and also significantly improved the survival rate (59).

A prospective case-control study investigated levels of HBD-2 in 16 patients with severe sepsis. HBD-2 plasma levels in septic patients were significantly higher compared to those in healthy controls and critically ill non-septic patients. Procalcitonin plasma levels and HBD-2 protein plasma levels showed a positive correlation in patients with severe sepsis. Moreover, the study investigated the *ex vivo* inducibility of HBD-2 mRNA in peripheral whole blood cells from patients with severe sepsis compared to non-septic critically ill patients and healthy individuals. Endotoxin-inducible HBD-2 mRNA expression was significantly decreased in patients with severe sepsis compared to healthy controls and non-septic critically ill patients, which may contribute to the complex immunological dysfunction in patients with severe sepsis. The contradiction between the decreased inducibility of HBD-2 in peripheral blood cells of patients with severe sepsis and the elevated levels of HBD-2 in septic plasma may suggest that in addition to peripheral blood cells, circulating endothelial cells or reticuloendothelial cells (e.g., monocytes or macrophages) may serve as a possible source of HBD-2 *in vivo* (60).

A prospective cross-sectional and longitudinal study in a university children’s hospital pediatric ICU investigated the systemic release of endogenous HNP 1–3 and lactoferrin in children with severe sepsis. Septic patients showed increased HNP 1–3 and lactoferrin plasma concentrations compared with non-septic critically ill control patients. Furthermore, HNP 1–3 and lactoferrin plasma concentrations correlated with total white blood cell and neutrophil counts. Although increased plasma lactoferrin concentrations were observed with the development of organ failure, there was no association between plasma HNP 1–3 concentration and organ failure or outcome. This observation is weakened by the fact that other mediators such as cytokines and nitrite radicals were not measured. Additionally, lactoferrin concentrations did not differ between non-survivors and survivors and did not correlate with the type of pathogen (61). The enhancement and adherence of neutrophils in damaged tissue may serve as a possible explanation for the correlation between lactoferrin concentration and organ failure. Moreover, high levels of lactoferrin were detected in patients with complement activation (62).

Another observational study determined HBD-2 levels and their impact on sepsis in term and preterm neonates at birth. HBD-2 levels in term neonates were higher compared with preterm infants and correlated with gestational age and birth weight. Of 31 preterm neonates, seven suffered from late-onset sepsis, and this was associated with lower HBD-2 levels (63). Furthermore, it was shown that HNP 1–3, lactoferrin, BPI, and heparin-binding protein (HBP) exerted higher levels in neonates with sepsis (64).

## Failed Attempts to Introduce AMP in Sepsis Therapy

Despite their discovery in 1939, AMPs are still rare in daily clinical practice. Currently, there are a significant number of products in development for topical applications of AMPs, such as BL 5010 (BiolineRX) against skin lesions or LTX-109 (Lytix) for the nasal eradication of *Staphylococcus aureus*.

To date, there have been only a few investigations into the therapeutic use of AMPs in sepsis. Promising results of the application of AMPs for meningococcal infection in children have been published (65). Children with suspected meningococcal sepsis were randomly assigned to receive a recombinant 21-kDA modified N-terminal fragment of human BPI (rBPI21) within 8 h of diagnosis (65). The administration of rBPI21 compared to placebo therapy was not superior with respect to mortality. One underlying reason may be the lower-than-expected placebo mortality (10% vs. expected 25%) because most deaths occurred in the interval between identification of patients and rBPI21 administration (65). However, children randomized to rBPI21 treatment showed a trend toward reduced multiple severe amputations and significantly higher physical and neurological abilities according to the pediatric overall performance category (POPC) scale (65).

Another AMP with clinical potential is the human lactoferrin peptide 1–11 (hLF 1–11), a derivative of the human lactoferrin that can be found in neutrophils or in body fluids (66). hLF 1–11 comprises antimicrobial activity and modulation of the inflammatory immune response. In a double-blind and placebo-controlled study to assess the side effects of hLF 1–11, the drug was tested in healthy volunteers and in patient undergoing hematopoietic stem cell transplantation. It showed a favorable side effect profile with only a slight elevation of liver enzymes (66). A planned study for the intravenous application of hLF 1–11 for 10 consecutive days in patients with bacteremia due to *Staphylococcus epidermidis* was withdrawn prior to enrollment for strategic reasons by AM pharma (NCT00509847). According to clinical trials, this decision was based on a strategic company decision. The homepage of AM pharma has no further information about hLF 1–11 or planned trials with the drug, so the future use of hLF 1–11 remains unclear (http://www.am-pharma.com).

**Figure 3:**
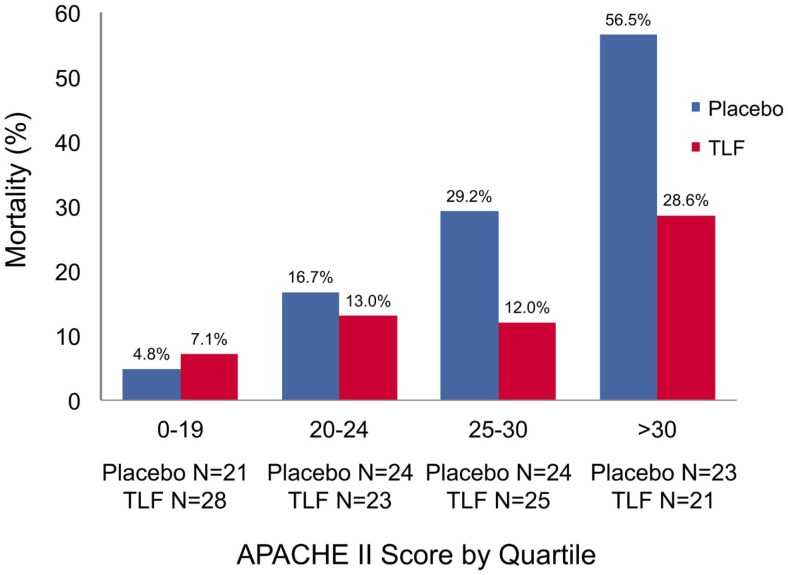
**Mortality of patients with severe sepsis and septic shock treated with talactoferrin or placebo**. The mortality is reported in relation to disease severity as expressed by the APACHE-II score. A positive effect of oral talactoferrin treatment on mortality in sepsis is detectable in patients with higher severity of disease (APACHE-II > 25). Patients with a lower APACHE-II score benefited less (APACHE-II 20–24) or not at all (APACHE-II < 19). APACHE-II score, Acute Physiology and Chronic Health Evaluation-II score. Reprinted with permission from Guntupalli et al. (67).

A different recombinant form of lactoferrin is talactoferrin alpha (TLF). TLF and lactoferrin possess identical molecular structures, biological activity and in other biochemical properties except for their nature of glycosylation (67). In a phase II study, 194 sepsis patients with at least one organ dysfunction were enrolled and assigned to a TLF or placebo group. Patients under medication with oral TLF showed a lower 28-day mortality rate with a sustained effect on mortality after 6 months. The decrease in mortality was more pronounced in patients with a higher severity of disease as expressed by APACHE-II scores above 25 points (Figure 3). Nonetheless, there was no significant difference regarding ICU days or ventilator-free days (67). Due to the promising results, a phase II/III study was initiated (safety and efficacy of TLF in patients with severe sepsis, OASIS; NCT 01273779). Surprisingly, the study was prematurely terminated due to the recommendation of the data safety monitoring board because of a higher 28-day mortality rate in the talactoferrin group. Here, the reason for failure remains unclear. One could speculate that oral administration is not the ideal route in critically ill patients who often suffer from gastroparesis and disturbed bowel motility.

The last attempt to use AMPs in sepsis was performed by stimulating cathelicidin levels through the administration of calcitriol in 67 patients with severe sepsis or septic shock (46). Though mRNA levels of cathelicidin increased, protein levels were comparable to a placebo group. The authors concluded that the dose and timing of calcitriol treatment might not have been ideally performed (68). Currently, no clinical trial on AMP treatment in sepsis has been initiated (clinicaltrials.gov; accession date February 10th, 2015).

## Future Aspects

The growing relevance of AMPs in recent years is because of their capacity to overcome increasing antibiotic resistance, which stems from their ability to decrease pro-inflammation, kill bacteria, and stimulate innate immunity (2–4). Whether one, two, or a combination of all three mechanisms will serve best is still a matter of debate. To date, the development of new drugs predominantly targets only single aspects of the body’s response to bacteria rather than bacterial pathogenicity factors (PF).

Synthetic AMPs based on the limulus-anti-LPS-factor (LALF) were designed to bind to the lipid A-moiety of LPS, thus decreasing inflammation and increasing survival in experimental sepsis (69). These synthetic AMPs exerted effectivity against Gram-negative bacteria and additionally against Gram-positive bacteria and mixed infections *in vitro* and in experimental settings *in vivo* (69–71). One further obstacle in the administration of AMPs is the application route. Previous trials used the local or oral route to administer AMPs in sepsis, with encouraging results in early studies. Phase 3 studies could not confirm the first trials (67). The application of designed AMPs is realized via continuous iv infusion and allows decreased inflammation (71), thereby improving the survival rate of septic mice (72). Moreover, virus attachment of enveloped viruses was shown to be decreased by a strong interaction between designed peptides and heparan sulfate (HS) (73). HS is a side chain shed in inflammation from proteoglycans (74, 75). Therefore, HS serves as a danger-associated molecular pattern (DAMP) and triggers a pro-inflammatory cascade in severe sepsis and septic shock (76). Peptide binding to and neutralization of HS may be the underlying mechanism for controlling inflammation (77). This prevents HS from binding to TLR-4, thus inhibiting TLR-4-downstream activation (78).

The combination of different AMPs with anti-inflammatory or bactericidal effects in varying doses may pave the way toward individualized therapy instead of a “one-size-fits-all” antibiotic attempt, which is the standard of care today. Moreover, AMPs offer the unique opportunity to cope with both DAMPs and pathogen-associated molecular patterns (PAMPs) such as LPS or lipopeptides (LPs). Due to this dual mechanism, designed AMPs exhibit their activity in both infectious *and* sterile inflammation (77). The underlying mechanism seems to be a charge-dependent alteration in the secondary structure that attenuates the inflammatory activities of DAMPs and PAMPs (69, 73). This ability distinguishes synthetic AMPs from conventional antibiotics and other attempts in sepsis therapy over the last several decades. Areas other than sepsis are currently the subjects of ongoing clinical studies to control inflammation; for example, talactoferrin, which failed in sepsis therapy (NCT01273779), is now being investigated in cancer studies (NCT00706862).

Thus, newly developed AMPs with decreased toxicity and a broad spectrum efficacy have the potential to improve therapy of bacterial and viral sepsis and to counter the increasing number of bacterial resistances against established antibiotics.

## Conflict of Interest Statement

Tobias Schuerholz received travel grants by Brandenburg Antiinfektiva GmbH. The remaining authors declare that the research was conducted in the absence of any commercial or financial relationships that could be construed as a potential conflict of interest.

## References

[1] Lehrer RI Ganz T Antimicrobial peptides in mammalian and insect host defence. J Surg Res (1999) 11:23–7. 10.1016/s0952-7915(99)80005-3 10047545

[2] Hamill P Brown K Jenssen H Hancock RE. Novel anti-infectives: is host defence the answer? Curr Opin Biotechnol (2008) 19:628–36. 10.1016/j.copbio.2008.10.006 19000763

[3] Hancock REW Sahl H-G. Antimicrobial and host-defense peptides as new anti-infective therapeutic strategies. Crit Care Med (2006) 24:1551–7. 10.1038/nbt1267 17160061

[4] Easton DM Nijnik A Mayer ML Hancock REW. Potential of immunomodulatory host defense peptides as novel anti-infectives. Trends Biotechnol (2009) 27(10):582–90. 10.1016/j.tibtech.2009.07.004 19683819 PMC7114281

[5] Ganz T Lehrer RI. Antimicrobial peptides of vertebrates. Curr Opin Immunol (1998) 10(1):41–4. 10.1371/journal.pone.0007358 9523109

[6] Jenssen H Hamill P Hancock REW. Peptide antimicrobial agents. Clin Microbiol Rev (2006) 19:491–511. 10.1128/CMR.00056-05 16847082 PMC1539102

[7] Kagan BL Ganz T Lehrer RI. Defensins: a family of antimicrobial and cytotoxic peptides. Toxicology (1994) 87:131–49. 10.1016/0300-483x(94)90158-9 7512758

[8] Yang D Biragyn A Hoover DM Lubkowski J Oppenheim JJ. Multiple roles of antimicrobial defensins, cathelicidins, and eosinophil-derived neurotoxin in host defense*. Annu Rev Immunol (2004) 22:181–215. 10.1146/annurev.immunol.22.012703.104603 15032578

[9] Zaiou M Gallo RL. Cathelicidins, essential gene-encoded mammalian antibiotics. J Mol Med (2002) 80:549–61. 10.1007/s00109-002-0350-6 12226737

[10] Wilmes M Sahl H-G. Defensin-based anti-infective strategies. Int J Med Microbiol (2014) 304:93–9. 10.1016/j.ijmm.2013.08.007 24119539

[11] Auvynet C Rosenstein Y. Multifunctional host defense peptides: antimicrobial peptides, the small yet big players in innate and adaptive immunity. FEBS J (2009) 276:6497–508. 10.1111/j.1742-4658.2009.07360.x 19817855

[12] Selsted ME Harwig SS. Purification, primary structure, and antimicrobial activities of a guinea pig neutrophil defensin. Infect Immun (1987) 55:2281–6. 10.1016/j.tibtech.2009.07.004 3623703 PMC260691

[13] Ganz T Selsted ME Szklarek D Harwig SS Daher K Bainton DF Defensins. Natural peptide antibiotics of human neutrophils. J Clin Invest (1985) 76:1427–35. 10.1172/JCI112120 2997278 PMC424093

[14] Zanetti M. The role of cathelicidins in the innate host defenses of mammals. Curr Issues Mol Biol (2005) 7:179–96. 16053249

[15] Chang TL Vargas J Jr DelPortillo A Klotman ME. Dual role of α-defensin-1 in anti-HIV-1 innate immunity. J Clin Invest (2005) 115:765–73. 10.1172/JCI21948 15719067 PMC548697

[16] Salvatore M García Sastre A Ruchala P Lehrer RI Chang T Klotman ME. α-defensin inhibits influenza virus replication by cell-mediated mechanism(s). J Infect Dis (2007) 196:835–43. 10.1086/521027 17703413

[17] Brown KL Hancock R. Cationic host defense (antimicrobial) peptides. Curr Opin Immunol (2006) 18:24–30. 10.1016/j.coi.2005.11.004 16337365

[18] Schneider JJ Unholzer A Schaller M Schäfer-Korting M Korting HC. Human defensins. J Mol Med (2005) 83:587–95. 10.1007/s00109-005-0657-1 15821901

[19] Rehaume LM Hancock REW. Neutrophil-derived defensins as modulators of innate immune function. Crit Rev Immunol (2008) 28:185–200. 10.1615/CritRevImmunol.v28.i3.10 19024344

[20] Kovach MA Standiford TJ. The function of neutrophils in sepsis. Curr Opin Infect Dis (2012) 25:321–7. 10.1097/QCO.0b013e3283528c9b 22421753

[21] O’Neill LAJ. Targeting signal transduction as a strategy to treat inflammatory diseases. Nat Rev Drug Discov (2006) 5:549–63. 10.1038/nrd2070 16773072

[22] Weighardt H Holzmann B. Role of toll-like receptor responses for sepsis pathogenesis. Immunobiology (2008) 212:715–22. 10.1016/j.imbio.2007.09.010 18086373

[23] Ciornei CD Sigurdardóttir T Schmidtchen A Bodelsson M. Antimicrobial and chemoattractant activity, lipopolysaccharide neutralization, cytotoxicity, and inhibition by serum of analogs of human cathelicidin LL-37. Antimicrob Agents Chemother (2005) 49:2845–50. 10.1128/AAC.49.7.2845-2850.2005 15980359 PMC1168709

[24] Sakoulas G Guram K Reyes K Nizet V Zervos M. Human cathelicidin LL-37 resistance and increased daptomycin MIC in methicillin-resistant Staphylococcus aureus strain USA600 (ST45) are associated with increased mortality in a hospital setting. J Clin Microbiol (2014) 52:2172–4. 10.1128/JCM.00189-14 24648548 PMC4042812

[25] Jeng L Yamshchikov AV Judd SE Blumberg HM Martin GS Ziegler TR Alterations in vitamin D status and anti-microbial peptide levels in patients in the intensive care unit with sepsis. J Transl Med (2009) 7:28–9. 10.1186/1479-5876-7-28 19389235 PMC2684740

[26] Liu PT Stenger S Li H Wenzel L Tan BH Krutzik SR Toll-like receptor triggering of a vitamin D-mediated human antimicrobial response. Science (2006) 311:1770–3. 10.1126/science.1123933 16497887

[27] Moromizato T Litonjua AA Braun AB Gibbons FK Giovannucci E Christopher KB. Association of low serum 25-hydroxyvitamin D levels and sepsis in the critically ill. Crit Care Med (2014) 42:97–107. 10.1097/CCM.0b013e31829eb7af 23982028

[28] Levy O. Antimicrobial proteins and peptides: anti-infective molecules of mammalian leukocytes. J Leukoc Biol (2004) 76:909–25. 10.1189/jlb.0604320 15292276

[29] Masson PL Heremans JF Metal-combining properties of human lactoferrin (red milk protein). 1. The involvement of bicarbonate in the reaction. Eur J Biochem (1968) 6:579–84. 10.1111/j.1432-1033.1968.tb00484.x 5701973

[30] Masson P Heremans JF Prignot J Immunohistochemical localization of the iron-binding protein lactoferrin in human bronchial glands. Experientia (1965) 21:604–5. 10.1007/BF02151559 5330250

[31] Vorland LH. Lactoferrin: a multifunctional glycoprotein. APMIS (1999) 107:971–81. 10.1111/j.1699-0463.1999.tb01499.x 10598868

[32] Chierici R Antimicrobial actions of lactoferrin. Adv Nutr Res (2001) 10:247–69. 10.1007/978-1-4615-0661-4_12 11795044

[33] Valenti P Marchetti M Superti F Amendolia MG Antiviral activity of lactoferrin. Adv Exp Med Biol (1998) 443:199–203. 10.1007/978-1-4757-9068-9 9781359

[34] Yen C-C Shen C-J Hsu W-H Chang Y-H Lin H-T Chen H-L Lactoferrin: an iron-binding antimicrobial protein against Escherichia coli infection. Biometals (2011) 24:585–94. 10.1007/s10534-011-9423-8 21327478

[35] Tomita M Wakabayashi H Shin K Yamauchi K Yaeshima T Iwatsuki K. Twenty-five years of research on bovine lactoferrin applications. Biochimie (2009) 91:52–7. 10.1016/j.biochi.2008.05.021 18585434

[36] Orsi N. The antimicrobial activity of lactoferrin: current status and perspectives. Biometals (2004) 17:189–96. 10.1023/B:BIOM.0000027691.86757.e2 15222464

[37] Zanetti M. Cathelicidins, multifunctional peptides of the innate immunity. J Leukoc Biol (2004) 75:39–48. 10.1189/jlb.0403147 12960280

[38] Braff MH Hawkins MA Nardo AD Lopez-Garcia B Howell MD Wong C Structure-function relationships among human cathelicidin peptides: dissociation of antimicrobial properties from host immunostimulatory activities. J Immunol (2005) 174:4271–8. 10.4049/jimmunol.174.7.4271 15778390

[39] Wilmes M Cammue BPA Sahl H-G Thevissen K. Antibiotic activities of host defense peptides: more to it than lipid bilayer perturbation. Nat Prod Rep (2011) 28:1350. 10.1039/c1np00022e 21617811

[40] Niyonsaba F Ushio H Nakano N Ng W Sayama K Hashimoto K Antimicrobial peptides human beta-defensins stimulate epidermal keratinocyte migration, proliferation and production of proinflammatory cytokines and chemokines. J Invest Dermatol (2007) 127:594–604. 10.1038/sj.jid.5700599 17068477

[41] Yang D Chertov O Bykovskaia SN Chen Q Buffo MJ Shogan J Beta-defensins: linking innate and adaptive immunity through dendritic and T cell CCR6. Science (1999) 286:525–8. 10.1126/science.286.5439.525 10521347

[42] Kondo T Takata H Takiguchi M Functional expression of chemokine receptor CCR6 on human effector memory CD8+ T cells. Intensive Care Med (2007) 37:54–65. 10.1002/eji.200636251 17171755

[43] Territo MC Ganz T Selsted ME Lehrer R. Monocyte-chemotactic activity of defensins from human neutrophils. J Clin Invest (1989) 84:2017–20. 10.1172/JCI114394 2592571 PMC304087

[44] Yang D Chen Q Chertov O Oppenheim JJ. Human neutrophil defensins selectively chemoattract naive T and immature dendritic cells. J Leukoc Biol (2000) 68:9–14. 10914484

[45] Froy O. Regulation of mammalian defensin expression by toll-like receptor-dependent and independent signalling pathways. Cell Microbiol (2005) 7:1387–97. 10.1111/j.1462-5822.2005.00590.x 16153239

[46] Lehrer RI Ganz T. Defensins of vertebrate animals. Curr Opin Immunol (2002) 14:96–102. 10.1016/S0952-7915(01)00303-X 11790538

[47] Stephens TA Nikoopour E Rider BJ Leon-Ponte M Chau TA Mikolajczak S Dendritic cell differentiation induced by a self-peptide derived from apolipoprotein E. J Immunol (2008) 181:6859–71. 10.4049/jimmunol.181.10.6859 18981105

[48] Lai Y Gallo RL. AMPed up immunity: how antimicrobial peptides have multiple roles in immune defense. Trends Immunol (2009) 30:131–41. 10.1016/j.it.2008.12.003 19217824 PMC2765035

[49] Scott MG Vreugdenhil AC Buurman WA Hancock RE Gold MR. Cutting edge: cationic antimicrobial peptides block the binding of lipopolysaccharide (LPS) to LPS binding protein. J Immunol (2000) 164:549–53. 10.4049/jimmunol.164.2.549 10623792

[50] Nijnik A Pistolic J Wyatt A Tam S Hancock REW. Human cathelicidin peptide LL-37 modulates the effects of IFN- on APCs. J Immunol (2009) 183:5788–98. 10.4049/jimmunol.0901491 19812202

[51] Mookherjee N Rehaume LM Hancock R. Cathelicidins and functional analogues as antisepsis molecules. Expert Opin Ther Targets (2007) 11(8):993–1004. 10.1517/14728222.11.8.993%20 17665972

[52] Semple F MacPherson H Webb S Cox SL Mallin LJ Tyrrell C Human β-defensin 3 affects the activity of pro-inflammatory pathways associated with MyD88 and TRIF. Intensive Care Med (2011) 41:3291–300. 10.1002/eji.201141648 21809339 PMC3494976

[53] Steinstraesser L Kraneburg UM Hirsch T Kesting M Steinau H-U Jacobsen F Host defense peptides as effector molecules of the innate immune response: a sledgehammer for drug resistance? Int J Mol Sci (2009) 10:3951–70. 10.3390/ijms10093951 19865528 PMC2769137

[54] Opal SM Palardy JE Marra MN Fisher CJ McKelligon BM Scott RW. Relative concentrations of endotoxin-binding proteins in body fluids during infection. Lancet (1994) 344:429–31. 10.1016/S0140-6736(94)91767-1 7520106

[55] Calvano SE Thompson WA Marra MN Coyle SM de Riesthal HF Trousdale RK Changes in polymorphonuclear leukocyte surface and plasma bactericidal/permeability-increasing protein and plasma lipopolysaccharide binding protein during endotoxemia or sepsis. Arch Surg (1994) 129:220–6. 10.1001/archsurg.1994.01420260116016 7508221

[56] Rintala E Peuravuori H Pulkki K Voipio-Pulkki LM Nevalainen T. Bactericidal/permeability-increasing protein (BPI) in sepsis correlates with the severity of sepsis and the outcome. Intensive Care Med (2000) 26:1248–51. 10.1007/s001340000616 11089749

[57] Ganz T. Defensins: antimicrobial peptides of innate immunity. Nat Rev Immunol (2003) 3:710–20. 10.1038/nri1180 12949495

[58] Schaller-Bals S Schulze A Bals R. Increased levels of antimicrobial peptides in tracheal aspirates of newborn infants during infection. Am J Respir Crit Care Med (2002) 165:992–5. 10.1164/ajrccm.165.7.200110-020 11934727

[59] Shu Q Shi Z Zhao Z Chen Z Yao H Chen Q Protection against *Pseudomonas aeruginosa* pneumonia and sepsis-induced lung injury by overexpression of beta-defensin-2 in rats. Shock (2006) 26:365–71. 10.1097/01.shk.0000224722.65929.58 16980883

[60] Book M Chen Q Lehmann LE Klaschik S Weber S Schewe J-C Inducibility of the endogenous antibiotic peptide beta-defensin 2 is impaired in patients with severe sepsis. Crit Care (2007) 11:R19. 10.1186/cc5694 17302973 PMC2151902

[61] Thomas NJ Carcillo JA Doughty LA Sasser H Heine RP. Plasma concentrations of defensins and lactoferrin in children with severe sepsis. Pediatr Infect Dis J (2002) 21:34–8. 10.1097/00006454-200201000-00008 11791096

[62] Wolach B Coates TD Hugli TE Baehner RL Boxer LA. Plasma lactoferrin reflects granulocyte activation via complement in burn patients. J Lab Clin Med (1984) 103:284–93. 6607301

[63] Olbrich P Pavón A Rosso ML Molinos A de Felipe B Sanchez B Association of human beta-defensin-2 serum levels and sepsis in preterm neonates*. Pediatr Crit Care Med (2013) 14:796–800. 10.1097/PCC.0b013e3182975e0f 23925144

[64] Berkestedt I Herwald H Ljunggren L Nelson A Bodelsson M. Elevated plasma levels of antimicrobial polypeptides in patients with severe sepsis. J Innate Immun (2010) 2:478–82. 10.1159/000317036 20571257

[65] Levin M Quint PA Goldstein B Barton P Bradley JS Shemie SD Recombinant bactericidal/permeability-increasing protein (rBPI21) as adjunctive treatment for children with severe meningococcal sepsis: a randomised trial. Lancet (2000) 356:961–7. 10.1016/S0140-6736(00)02712-4 11041396

[66] Van Der Velden WJ Van Iersel TM Blijlevens NM Donnelly JP. Safety and tolerability of the antimicrobial peptide human lactoferrin 1-11 (hLF1-11). BMC Med (2009) 7:44. 10.1186/1741-7015-7-44 19735580 PMC2746231

[67] Guntupalli K Dean N Morris PE Bandi V Margolis B Rivers E A phase 2 randomized, double-blind, placebo-controlled study of the safety and efficacy of talactoferrin in patients with severe sepsis*. Crit Care Med (2013) 41:706–16. 10.1097/CCM.0b013e3182741551 23425819

[68] Leaf DE Raed A Donnino MW Ginde AA Waikar SS. Randomized controlled trial of calcitriol in severe sepsis. Am J Respir Crit Care Med (2014) 190:533–41. 10.1164/rccm.201405-0988OC 25029202 PMC4214090

[69] Gutsmann T Razquin-Olazarán I Kowalski I Kaconis Y Howe J Bartels R New antiseptic peptides to protect against endotoxin-mediated shock. Antimicrob Agents Chemother (2010) 54:3817–24. 10.1128/AAC.00534-10 20606063 PMC2934961

[70] Heinbockel L Palacios-Chaves L Alexander C Rietschel E Behrends J Correa W Mechanism of Hbγ-35-induced an increase in the activation of the human immune system by endotoxins. Innate Immun (2015) 21:305–13. 10.1177/1753425914535957 25034969

[71] Schuerholz T Doemming S Hornef M Martin L Simon T-P Heinbockel L The anti-inflammatory effect of the synthetic antimicrobial peptide 19-2.5 in a murine sepsis model: a prospective randomized study. Crit Care (2013) 17:R3. 10.1186/cc11920 23302299 PMC4057408

[72] Heinbockel L Sanchez-Gómez S Martinez-De-Tejada G Dömming S Brandenburg J Kaconis Y Preclinical investigations reveal the broad-spectrum neutralizing activity of peptide pep19-2.5 on bacterial pathogenicity factors. Antimicrob Agents Chemother (2013) 57:1480–7. 10.1128/AAC.02066-12 23318793 PMC3591871

[73] Krepstakies M Lucifora J Nagel C-H Zeisel MB Holstermann B Hohenberg H A new class of synthetic peptide inhibitors block attachment and entry of human pathogenic viruses. J Infect Dis (2012) 205:1654–64. 10.1093/infdis/jis273 22457281

[74] Parish CR The role of heparan sulphate in inflammation. Nat Rev Immunol (2006) 6:633–43. 10.1038/nri1918 16917509

[75] Li J-P Vlodavsky I. Heparin, heparan sulfate and heparanase in inflammatory reactions. Thromb Haemost (2009) 102:823–8. 10.1160/TH09-02-0091 19888515

[76] Johnson GB Brunn GJ Kodaira Y Platt JL. Receptor-mediated monitoring of tissue well-being via detection of soluble heparan sulfate by toll-like receptor 4. J Immunol (2002) 168:5233–9. 10.4049/jimmunol.168.10.5233 11994480

[77] Martin L Schmitz S De Santis R Doemming S Haase H Hoeger J Peptide 19-2.5 inhibits heparan sulfate-triggered inflammation in murine cardiomyocytes stimulated with human sepsis serum. PLoS One (2015) 10:e0127584. 10.1371/journal.pone.0127584 26024383 PMC4449035

[78] Goodall KJ Poon IKH Phipps S Hulett MD. Soluble heparan sulfate fragments generated by heparanase trigger the release of pro-inflammatory cytokines through TLR-4. PLoS One (2014) 9:e109596. 10.1371/journal.pone.0109596 25295599 PMC4190175

